# Nutritional Intake in Oropharyngeal Dysphagia: A Retrospective Comparison of Traditional Homogenized and Density-Enriched Prepared Diets

**DOI:** 10.3390/foods15122104

**Published:** 2026-06-11

**Authors:** Marco Cintoni, Elena Leonardi, Pauline Celine Raoul, Giorgia Buscaino, Marta Palombaro, Emanuele Rinninella, Esmeralda Capristo, Antonio Gasbarrini, Maria Cristina Mele

**Affiliations:** 1 UOC di Nutrizione Clinica, Dipartimento di Scienze Mediche e Chirurgiche, Fondazione Policlinico Universitario A. Gemelli IRCCS, Largo A. Gemelli 8, 00168 Rome, Italy; elena.leonardi2@unicatt.it (E.L.); marta.palombaro@guest.policlinicogemelli.it (M.P.); emanuele.rinninella@unicatt.it (E.R.); mariacristina.mele@unicatt.it (M.C.M.); 2 Centro di Ricerca e Formazione in Nutrizione Umana, Università Cattolica del Sacro Cuore, 00168 Rome, Italy; esmeralda.capristo@unicatt.it (E.C.); antonio.gasbarrini@unicatt.it (A.G.); 3 Scuola di Specializzazione in “Scienza dell’Alimentazione”, Università Cattolica del Sacro Cuore, 00168 Rome, Italy; giorgia.buscaino01@icatt.it; 4 Dipartimento di Medicina e Chirurgia Traslazionale, Università Cattolica del Sacro Cuore, 00168 Rome, Italy; 5 UOS Medicina della Grande Obesità, Dipartimento di Scienze Mediche e Chirurgiche, Fondazione Policlinico Universitario A. Gemelli IRCCS, Largo A. Gemelli 8, 00168 Rome, Italy; 6 UOC Medicina Interna e Gastroenterologia, Dipartimento di Scienze Mediche e Chirurgiche, Fondazione Policlinico Universitario A. Gemelli IRCCS, Largo A. Gemelli 8, 00168 Rome, Italy

**Keywords:** dysphagia, texture-modified diet, density-enrichment, IDDSI, nutritional intake, protein density, hospital nutrition, palatability

## Abstract

Oropharyngeal dysphagia is prevalent in hospitalized geriatric and neurological populations and constitutes a major driver of disease-related malnutrition. Conventional texture-modified diets frequently rely on diluting solid foods with liquid agents to achieve safe swallowing consistency, a process that reduces caloric and protein density per gram and creates a so-called volume paradox, whereby large meal volumes deliver inadequate nutrients. This retrospective observational study, conducted at the Fondazione Policlinico Gemelli IRCCS in Rome, compared nutritional intake in 208 hospitalized dysphagic adults receiving either a traditional homogenized standard diet (THSD; n = 58) or a density-enriched dysphagia-prepared diet (DPD; n = 150). Following propensity-score matching, total daily energy intake was significantly higher with the DPD compared to the THSD (1024 ± 307 kcal vs. 523 ± 161 kcal; *p* < 0.0001), as was total protein intake (37.3 ± 12.9 g vs. 26.2 ± 12.7 g; *p* < 0.0001). Clinically meaningful differences were observed across all meal components, including a more than twofold advantage in breakfast protein content (6.6 ± 1.7 g vs. 3.0 ± 1.5 g). Despite these improvements, total energy and protein intake remained below estimated daily requirements in both groups, highlighting the need for systematic nutritional monitoring alongside catering optimization. These findings support density-enrichment as a practical and safe strategy for improving nutritional adequacy in dysphagic inpatients, with implications for reducing reliance on oral nutritional supplements and mitigating disease-related malnutrition in clinical settings.

## 1. Introduction

Oropharyngeal dysphagia represents a pervasive clinical challenge, characterized by impaired deglutition and affecting more than 30% of patients in geriatric and neurological settings [1,2]. This condition often arises from both acute and chronic medical issues, including neurological disorders, cancer, and age-related physiological changes, thereby rendering patients vulnerable to a cascade of complications [3,4]. Given this widespread occurrence, it becomes necessary to integrate effective screening and management strategies within clinical pathways to address and treat dysphagia adequately.

The link between dysphagia and disease-related malnutrition (DRM) is well established [5]: impaired chewing and swallowing directly reduce caloric and protein intake, with particular consequences for older adults who already face elevated nutritional demands alongside illness-related anorexia and fatigue [6,7]. Malnourished patients show poorer recovery, higher rates of infection, and longer hospital stays, [6,8,9], underscoring the need for integrated nutritional management within dysphagia care pathways [1,10].

In response to these nutritional challenges, dietary texture modification has become a standard clinical protocol for patients with dysphagia in hospital settings. Within this framework, the preparation of homogenized meals requires mechanical rheological modification of standard food items to achieve a cohesive, lump-free bolus, primarily achieved through the dilution of solid food matrices with liquid agents, such as water, broth, or milk, to meet the viscosity requirements necessary for safe oropharyngeal transit, and substantial modifications to standard food items to achieve a safe, lump-free consistency [11]. This process aims to make the food more palatable and easier to swallow for individuals with oropharyngeal dysphagia. However, these additions can lead to nutrient dilution, potentially compromising the dietary quality of the modified foods [12]. It is essential to note that while safety in swallowing is prioritized, the nutritional adequacy of these diets can be adversely affected [13]. This dilution can lead to the so-called “volume paradox.” As liquids are incorporated to create palatable purees or soups, the overall volume of the meal increases significantly. However, the caloric and protein density per gram may decrease considerably [12]. The high volume of low-density meals may not provide the necessary energy and protein essential for recovery, especially for dysphagic patients who already struggle with maintaining adequate nutritional intake [14].

Advanced texture-modified diets are specialized nutritional strategies designed to address the inherent limitations of traditional homogenized diets. This innovative approach utilizes enriched lyophilized products, providing a solution that ensures safety in consumption while enhancing nutrient intake [15]. By focusing on the nutritional density of the meals, these products allow for significant intake of essential nutrients without the excessive volume typically associated with texture-modified diets [16]. A critical component of these products is their high nutritional density, achieved through systematic enrichment of protein and calories across all meals, including breakfast, lunch, and dinner. This methodology enables healthcare providers to formulate diets that meet energy requirements while maintaining smaller meal volumes, thereby mitigating early satiety, which often hinders frail patients from reaching their nutritional targets [17,18]. Each meal is meticulously crafted to ensure that even in reduced servings, the caloric and protein contents remain adequate, thus supporting the overall nutritional health of dysphagic patients [18]. Safety in food preparation and consumption is paramount in dysphagia management. These products adhere strictly to the International Dysphagia Diet Standardization Initiative (IDDSI) levels, which guarantee consistency in food texture and create a framework for clinicians to follow [19]. This standardization is crucial in preventing aspiration pneumonia, a serious complication that can arise from mismanaged dysphagia diets [20,21]. In this context, considering the well-established correlations between dysphagia, malnutrition, and adverse clinical outcomes, optimizing hospital catering models is an important intervention strategy.

To date, evidence comparing density-enriched and traditional texture-modified diets in real-world inpatient settings remains limited, with most published data derived from small-scale pilot studies or non-clinical populations; the present study addresses this gap by providing large-scale, hospital-based comparative data with rigorous propensity-score adjustment.

Hence, this retrospective study aims to compare adherence to two hospital texture-modified diets adapted for patients with dysphagia: a traditional homogenized standard diet (THSD) and a dysphagia-prepared diet (DPD).

## 2. Materials and Methods

### 2.1. Study Design

An observational study was conducted among hospitalized patients in the medical wards at the Fondazione Policlinico Gemelli IRCCS in Rome from August 2025 to January 2026. The study was conducted in accordance with the Helsinki Declaration and Good Clinical Practice and was approved by the Ethics Committee of our institution (ID 6935). Informed consent was obtained from all study participants.

### 2.2. Objectives

This study aims to assess adherence to the two dietary types by conducting a retrospective comparative analysis of nutritional intake data. The primary objective is to compare dietary compliance among patients with dysphagia. The secondary outcomes are macronutrients’ daily intake, specifically energy and protein, to quantify the gap between estimated dietary requirements and actual consumption intake across the two distinct modified diets.

### 2.3. Characteristics of Diets

Patients were retrospectively categorized according to the diet received: the DPD or the THSD. The THSD is a balanced diet consisting of semolina, blended proteins (fish, meat), and vegetable cream soup—all composed of natural foods with smooth consistency (IDDSI Level 4, Puréed)—and fruit mousse. The food is mechanically modified with liquid agents (water, broth, or milk) to achieve a lump-free consistency without additional nutritional enrichment. The breakfast is composed of yogurt mixed with a soluble biscuit. The DPD consists of meals in which food technology is used to achieve personalized levels of density, viscosity, texture, and particle size, also targeting IDDSI Level 4 (Puréed) consistency—the same swallowing-safety threshold as the THSD. Some Italian recipes use traditional flavours that are condensed, dried, and packaged for later use. The food is thickened with proteins or naturally occurring bulking agents (e.g., collagen), rather than with additional natural thickeners such as potatoes.

The DPD (HARG SB Srl, Brescia, Italy) is produced under standardised industrial conditions, ensuring batch-to-batch consistency; the collagen-based binding protocol allows compact meal volumes to meet IDDSI Level 4 rheological requirements without liquid dilution. The DPD additionally includes a dessert component at each midday and evening service, deliberately incorporated as part of the density-enrichment strategy to distribute caloric and protein load across a greater number of eating occasions and mitigate early satiety in frail patients; this structural difference between the two catering protocols is reflected in Table 1.

The protein and energy content of each meal for the two diet types is presented in Table 1.

### 2.4. Data Collection

Patients were selected by trained dietitians from the Clinical Nutrition Unit by using the hospital’s in-house patient information system. The inclusion criteria were adult patients aged 18 years and above who received the traditional homogenized standard diet or the dysphagia-prepared diet orally during their hospital stay. Exclusion criteria included those who were on enteral or parenteral feeding, had mental problems or disorders, or were diagnosed with dementia, or those who did not agree to participate in the study and thus did not sign the consent form. Sociodemographic data and accurate food intake have been collected. In addition to the data required for inclusion, our datasets included information from patients’ previous medical records. The indicators used in the present study included routinely collected data, such as body weight, height, and ONS supplementation.

Our questionnaire is consistent with established practices in clinical nutrition and foodservice research. Indeed, comparable methodologies have been adopted in prior studies, including those by Naithani et al. [17], Williams and Walton [18], and Mills et al. [19], supporting the appropriateness of using context-specific instruments in hospital-based investigations.

Because the food-weighing method was infeasible due to its time-consuming and labor-intensive nature, the visual estimation method, expressed on a 5-point scale, was chosen to measure the percentage of food intake. Throughout the day (across breakfast, lunch, and dinner), food intake—separated into different food components (first course, second course, vegetables, fruit, and dessert)—was observed by trained clinical staff and recorded in values ranging from 0%, 25%, 50%, 75%, to 100% (none consumed to all consumed). Successively, food intake percentages were converted into estimated caloric and protein values using the nutritional reference data from the Hospital Diet Manual. Each meal component was assigned a fixed nutritional value, and the observed intake percentage for each component was multiplied by its corresponding energy and protein content to determine the actual intake. To estimate patients’ minimum nutritional requirements, average basal metabolic rates for men and women, as well as protein requirements, were calculated using the Reference Intake of Nutrients and Energy for the Italian Population (LARN) [22]. The specific questionnaire consists of questions that evaluate the food quality domain (integrity of trays/plates/cutlery, odour, temperature, palatability, provision quantity, degree of cooking, menu variety). The scoring criteria used in this questionnaire were a 5-point scale (insufficient, sufficient, good, very good, not applicable).

### 2.5. Statistical Analysis

Continuous variables were assessed for normality and reported as the mean ± standard deviation (SD). To rigorously evaluate the discrepancies in macronutrient delivery (energy and protein) between THSD and DPD cohorts, independent samples Student’s *t*-tests were deployed with between-group comparisons (THSD vs. DPD) performed both before and after propensity-score matching. Given the retrospective observational nature of the study design, a propensity-score matching algorithm was proactively applied to mitigate inherent selection biases and strictly control for confounding baseline demographic covariates (specifically, age and sex) between the two intervention groups. The threshold for statistical significance was rigorously defined at an alpha level of *p* < 0.05. All statistical analysis and matching procedures were executed utilizing STATA software (version 18.0).

## 3. Results

### 3.1. Characteristics of Patients

The present retrospective analysis included a cohort of 208 adult inpatients diagnosed with oropharyngeal dysphagia. The study population was predominantly admitted to medical wards (79.3%), with the remainder in surgical settings (20.7%). The sample comprised 58 patients (27.8%) allocated to THSD and 150 patients (72.2%) receiving DPD. The mean age of the overall cohort was 66.7 ± 8.2 years, with a comparable sex distribution across both dietetic models (females accounted for approximately 42% of the population).

### 3.2. Comparative Analysis of Energy and Protein Intake

Table 2 summarizes the energy and protein intake across the two diets.

Unadjusted descriptive data indicated a mean total energy intake of 523 ± 161 kcal/day for the THSD group versus 990 ± 289 kcal/day for the DPD group, alongside protein intakes of 26.2 ± 12.7 g/day and 36.0 ± 12.2 g/day, respectively. To rigorously control for potential baseline confounders, a propensity-score matching procedure adjusting for age and sex was implemented, yielding two highly comparable cohorts of 58 patients each. Subsequent statistical evaluation of the matched cohorts demonstrated that the DPD intervention resulted in a significantly superior macronutrient delivery across all evaluated parameters. Specifically, the mean total energy intake in the DPD group was 1024 ± 307 kcal, significantly higher than the 523 ± 161 kcal observed in the THSD arm (*p* < 0.0001). Concurrently, total protein consumption was significantly increased in the DPD group compared with the THSD group (37.3 ± 12.9 vs. 26.2 ± 12.7 g; *p* < 0.0001). An analysis of consumption efficiency—defined as the ratio of actual intake to provisioned nutrients—revealed that patients receiving the DPD consumed 57.3% of the dietary energy provided by the catering system (1024 of 1786.1 kcal offered). Conversely, the THSD cohort exhibited a consumption efficiency of merely 42.2% (523 of 1239 kcal). This enhanced nutritional intake was consistently observed across distinct meal components; notably, the DPD facilitated a more than twofold increase in breakfast protein yield (6.6 ± 1.7 vs. 3.0 ± 1.5 g) and delivered significantly higher caloric content during both the first (281 ± 96 vs. 200 ± 127 kcal) and second courses (258 ± 89 vs. 82 ± 57 kcal).

### 3.3. Comparative Analysis of Meal Quality Perception

Table 3 summarizes the meal quality characteristics evaluated by patients for both diets.

Across all courses, the DPD achieved a significantly higher percentage of positive ratings than the THSD. During breakfast, patients in the DPD group reported higher scores for appetibility (82.76% vs. 67.12%; *p* = 0.026) and quantity adequacy (91.38% vs. 73.33%; *p* = 0.005). The same trend continued into the first course, where the DPD was rated significantly higher across all evaluated dimensions, including appetibility (68.97% vs. 31.33%; *p* < 0.001), degree of doneness (94.83% vs. 41.33%; *p* < 0.001), temperature (86.21% vs. 48.99%; *p* < 0.001), and quantity (93.10% vs. 50.00%; *p* < 0.001). Similarly, for the second course, the DPD model consistently outperformed the traditional group in degree of doneness (94.83% vs. 41.33%; *p* < 0.001), smell (53.45% vs. 19.33%; *p* < 0.001), appetibility (72.41% vs. 20.67%; *p* < 0.001), temperature (98.28% vs. 54.00%; *p* < 0.001), and quantity (92.98% vs. 39.33%; all *p* < 0.001). A comparable pattern was observed for the vegetable side dishes, which yielded higher ratings in the DPD group for temperature (98.18% vs. 46.00%; *p* < 0.001), smell (70.91% vs. 20.00%; *p* < 0.001), appetibility (69.09% vs. 25.33%; *p* < 0.001), degree of doneness (96.36% vs. 40.00%; *p* < 0.001), and quantity (96.23% vs. 39.33%; *p* < 0.001). Finally, regarding the fruit portion, quantity adequacy was rated positively by 94.55% of the DPD group compared to only 50.00% of the THSD group (*p* < 0.001).

## 4. Discussion

This retrospective study compared nutritional intake (energy and protein) between a THSD and a density-enriched DPD, demonstrating that the DPD ensures better energy and protein intake than the THSD. Specifically, the DPD group achieved nearly double the daily caloric intake and significantly higher protein consumption. This nutritional advantage was evident across meals, with breakfast providing more than twice the protein and the first course yielding substantially higher energy. These results highlight the superior nutrient density of the DPD. However, despite these significant improvements, total nutrient intake in both groups remained below the estimated daily requirements for hospitalized adults. This persistent deficit underscores the critical need for systematic nutritional screening and proactive clinical protocols to bridge the gap between actual oral consumption and individual metabolic demands. These data align with evidence suggesting that density enrichment within texture-modified diets enhances nutrient delivery without compromising swallow safety, and that improvements in taste and presentation can boost intake when textures are engineered for both density and palatability [16,23,24,25,26].

The central finding can be explained through the comparative mechanics of texture modification. The THSD relies on mechanical rheological modification—often diluting solid food with liquids—to achieve IDDSI-compatible safety. Consistent with the broader literature, traditional dilution-based modification yields lower energy and protein intake unless specific enrichment strategies are employed, whereas density-enrichment facilitates higher intake without requiring large meal volumes [16,27,28]. Consequently, the higher daily energy and protein intake observed with the DPD reflects the practical advantage of its density-focused formulation. From a food-technology perspective, the DPD exemplifies a density-first paradigm. By utilizing enriched, freeze-dried components and natural binders (such as collagen) instead of starch or liquid diluents, it enables safe, IDDSI Level 4 textures within compact meal volumes. This paradigm is well-described in various studies as a means to reconcile swallow safety with nutrient adequacy, with preliminary data suggesting improved intake, reduced supplementation, and reduced waste when high-density formulations are implemented within IDDSI-compliant frameworks [29,30,31,32]. Furthermore, sensory optimization—such as the incorporation of familiar Italian flavour profiles and appealing processing in the DPD—appears to enhance appetite and adherence. Reviews consistently show that palatability, aroma, and appearance heavily influence intake, and that enrichment yields better mealtime satisfaction without compromising safety [18,25,26]. Clinically, the protein advantage observed with the DPD is highly meaningful. Protein plays a crucial role in supporting lean mass, functional recovery, and sarcopenia prevention in older adults with dysphagia or neurodegenerative conditions [33,34,35,36]. The 11 g of daily protein advantage, combined with the breakfast gain of 3.6 g, has significant implications for nitrogen balance and muscle preservation. Because protein is a rate-limiting nutrient for recovery in geriatric populations, utilizing protein-dense textures and distributing them strategically across meals is essential to sustain anabolic signalling [24,26,34,35]. Additionally, safety, standardization, and the broader healthcare burden constitute another critical axis. IDDSI standardization improves swallow-safety communication and reduces misclassification, enabling reliable nutrition delivery across varying care settings [28,29,37,38].

Regarding protein quality, the DPD relies primarily on collagen as a binding agent. While the total protein advantage of the DPD over the THSD is clinically meaningful, future studies should characterise the amino acid profile of the protein sources employed—particularly the content of essential and branched-chain amino acids, given their central role in muscle protein synthesis and anabolic signalling in geriatric populations [33,34,35]. Furthermore, the use of hydrocolloids with dual thickening and lubrication properties, such as agar, gellan gum, or mucoadhesive polysaccharides, represents a promising avenue for further optimising texture-modified diets; such agents could simultaneously ensure IDDSI-compliant consistency and facilitate bolus transit, warranting investigation in future formulation studies.

A finding of utmost clinical relevance that strongly emerges from this analysis is that, despite the significant improvements achieved with the DPD compared to the THSD, total nutritional intake in both groups remained substantially below the estimated daily requirements for hospitalized patients. Reaching an average of only 1024 kcal and 37 g of protein per day represents an excessively low—and somewhat alarming—value that fails to provide adequate metabolic and anabolic support, particularly for frail populations or those with neurological conditions. The multifactorial nature of this persistent deficit warrants explicit consideration: disease-related anorexia, fatigue, swallowing-related fear and anxiety (deglutophobia), early satiety secondary to reduced gastric motility, and the hypermetabolic demands of acute illness and neurological conditions all contribute to suboptimal oral intake in this population. The downstream consequences are equally serious: accelerated lean mass loss, sarcopenia progression, impaired immune function, and prolonged hospitalization are all directly linked to protein–energy malnutrition in geriatric and neurological inpatients [6,8,33,34,35]. This persistent nutritional deficit underscores that protein-energy malnutrition remains an ongoing clinical challenge that is directly correlated with higher morbidity and mortality rates and prolonged hospital stays. These results highlight that while technological optimization of hospital catering via density enrichment is an effective strategy, it cannot be considered a standalone solution. Therefore, comprehensive multidisciplinary management by the Clinical Nutrition service is fundamental. Physicians and dietitians must continuously monitor actual intake to promptly tailor the diet to individual patient needs and evaluate the targeted introduction of ONS to bridge the critical gap between spontaneous oral consumption and actual metabolic demands.

It should be noted that part of the caloric and protein advantage of the DPD is attributable to the additional dessert component absent from the THSD. However, even when the dessert contribution is subtracted from the DPD total (approximately 62 kcal and 4.1 g protein), the DPD group still achieves substantially higher energy (approximately 962 kcal vs. 523 kcal) and protein intake (approximately 33.2 g vs. 26.2 g) than the THSD group, confirming that the nutritional advantage is not solely driven by this additional meal component.

Beyond improving nutritional intake, adopting DPD could offer significant opportunities to reduce food waste and optimize the use of economic resources. Uneaten food in healthcare facilities represents a major critical issue, with waste volumes ranging from 17% to 67% depending on the foodservice system implemented [39]. This phenomenon has severe repercussions for sustainability and the environment, as well as significant economic losses for the healthcare system. The recent literature highlights that optimizing meal presentation and improving the texture of modified diets are effective strategies to significantly decrease plate waste while simultaneously increasing patient satisfaction. Therefore, the higher acceptance and excellent portion adequacy observed with the DPD could not only facilitate clinical recovery but also reduce the need to rely on costly fortified ONS systematically. A targeted dietary approach, supported by specialized personnel, thus translates into a potential reduction in overall hospital costs associated with waste, promoting a model of care that is clinically effective, ecologically sustainable, and economically viable.

Several limitations of this study must be acknowledged. Given its retrospective and single-center design, causal relationships cannot be definitively established. Potential confounders, such as baseline functional status or concurrent rehabilitation interventions, may have influenced nutritional intake. In addition, formal dysphagia severity grading (e.g., Functional Oral Intake Scale or videofluoroscopic swallowing study classification) was not systematically collected in this retrospective dataset and should be regarded as a residual confounding variable; future prospective studies should capture this measure to allow for severity-stratified analyses. Furthermore, nutritional intake data were collected via visual estimation by trained clinical staff. While pragmatic for a hospital setting, this method lacks the absolute precision of direct food-weighing and is susceptible to observer bias. This limitation is, however, likely non-differential between the two diet groups, as both were assessed by the same clinical staff using the same standardised protocol; a systematic directional bias in the between-group comparison is therefore unlikely, though the possibility cannot be entirely excluded. Future research should prioritize multicenter, prospective randomized controlled trials (RCTs) to confirm the causal relationship between density-enriched diets and improvements in nutritional status. Moreover, while this study relied on questionnaires completed by clinical professionals, subsequent trials should incorporate objective physical assessments (e.g., bioelectrical impedance analysis or handgrip strength) to quantify the impact of the DPD on muscle recovery and function. Finally, long-term clinical outcomes—such as the reduction in aspiration pneumonia rates, length of hospitalization, and the potential decrease in hospital ONS use—warrant further investigation.

## 5. Conclusions

The transition from a traditional, dilution-based food modification diet to a density-enriched diet represents a significant advancement in the nutritional management of hospitalized patients with dysphagia. The application of food technology—specifically through the use of protein-based binders and enriched formulations—successfully addresses the “volume paradox” by delivering high-density nutrition within manageable meal portions. This approach significantly enhances patients’ ability to meet their energy and protein requirements without exceeding their physical or sensory tolerances. Furthermore, systematic evaluation and clinical management by the Clinical Nutrition Units remain fundamental to tailoring the hospital diet to individual patient needs and to integrating ONS as needed. Adopting standardized, IDDSI-compliant enriched diets could be a practical and effective strategy for mitigating DRM in clinical settings.

## Figures and Tables

**Table 1:** Nutritional characteristics of the two texture-modified diets.

		Breakfast	First Course	Second Course	Vegetables	Fruit	Dessert	Total Daily Intake *
THSD	Energy (kcal)	179	245	115	80	90	-	1239
DPD		170.6	229.7	210.8	225.4	90.7	102.3	1786.1
THSD	Proteins (g)	5.4	7	23	4	<0.5 **	-	74.4
DPD		7.7	6.8	7.3	7.2	8.4	6.9	74

* The total daily intake was calculated considering the sum of one breakfast, two first courses, two second courses, two vegetable portions, and two servings of fruit; for the DPD, one dessert was also included in the calculation. ** <0.5 g denotes a sub-threshold estimate; fruit in the THSD does not contain a discrete protein source. Abbreviations: DPD, Dysphagia prepared diet; THSD, Traditional Homogenized Standard Diet.

**Table 2:** (a) Calculated average consumed energy (kcal) by meal component—THSD vs. DPD, before and after propensity-score matching. (b) Calculated average consumed protein (g) by meal component—THSD vs. DPD, before and after propensity-score matching.

**(a)**						
	**Before Matching**			**After Matching**		
**Meal Component (Mean ± SD)**	**THSD (n = 58) kcal**	**DPD (n = 150) kcal**	***p***	**THSD (n = 58) kcal**	**DPD (n = 58) kcal**	***p***
Breakfast	101 ± 49	143 ± 36	<0.0001	101 ± 49	148 ± 37	<0.0001
First course	200 ± 127	261 ± 106	<0.0001	200 ± 127	281 ± 96	<0.0001
Second course	82 ± 57	251 ± 98	<0.0001	82 ± 57	258 ± 89	<0.0001
Vegetables	40 ± 22	164 ± 126	<0.0001	40 ± 22	161 ± 128	<0.0001
Fruit	98 ± 56	109 ± 65	0.04	98 ± 56	113 ± 65	<0.0001
Dessert	−	62 ± 37		−	64 ± 37	
Total	523 ± 161	990 ± 289	<0.0001	523 ± 161	1024 ± 307	<0.0001
**(b)**						
	**Before Matching**			**After Matching**		
**Meal Component (Mean ± SD)**	**THSD (n = 58) g Protein**	**DPD (n = 150) g Protein**	***p***	**THSD (n = 58) g Protein**	**DPD (n = 58) g Protein**	***p***
Breakfast	3.0 ± 1.5	6.4 ± 1.6	<0.0001	3.0 ± 1.5	6.6 ± 1.7	<0.0001
First course	5.7 ± 3.6	7.7 ± 3.1	<0.0001	5.7 ± 3.6	8.3 ± 2.8	<0.0001
Second course	16.5 ± 11.4	9.9 ± 3.8	<0.0001	16.5 ± 11.4	10.2 ± 3.5	<0.0001
Vegetables	4.0 ± 2.2	4.3 ± 3.2	0.05	4.0 ± 2.2	4.2 ± 3.3	0.08
Fruit	0 ± 0	10.0 ± 6.1	<0.0001	0 ± 0	10.4 ± 6.0	<0.0001
Dessert	−	4.1 ± 2.5		−	4.3 ± 2.5	
Total	26.2 ± 12.7	36.0 ± 12.2	<0.0001	26.2 ± 12.7	37.3 ± 12.9	<0.0001

*p* < 0.0001 vs. THSD within the same matching condition (independent samples *t*-test). Statistics not performed: no dessert component in the THSD. Abbreviations: DPD, Dysphagia Prepared Diet; THSD, Traditional Homogenized Standard Diet; SD, standard deviation.

**Table 3:** Association between diet type (DPD vs. THSD) and meal quality criteria.

	DPD (n = 150)	THSD (n = 58)	*p*-Value
Breakfast			
Appetibility (% Good)	82.76%	67.12%	0.026
Quantity (% Good)	91.38%	73.33%	0.005
First Course			
Appetibility (% Good)	68.97%	31.33%	<0.001
Degree of Doneness (% Correct)	94.83%	41.33%	<0.001
Temperature (% Correct)	86.21%	48.99%	<0.001
Quantity (% Good)	93.10%	50.00%	<0.001
Second Course			
Degree of Doneness (% Correct)	94.83%	41.33%	<0.001
Smell (% Good)	53.45%	19.33%	<0.001
Appetibility (% Good)	72.41%	20.67%	<0.001
Temperature (% Correct)	98.28%	54.00%	<0.001
Quantity (% Good)	92.98%	39.33%	<0.001
Vegetables			
Temperature (% Correct)	98.18%	46.00%	<0.001
Smell (% Good)	70.91%	20.00%	<0.001
Appetibility (% Good)	69.09%	25.33%	<0.001
Degree of Doneness (% Correct)	96.36%	40.00%	<0.001
Quantity (% Good)	96.23%	39.33%	<0.001
Fruit			
Quantity (% Good)	94.55%	50.00%	<0.001

Abbreviations: DPD, Dysphagia prepared diet; THSD, Traditional Homogenized Standard Diet.

## Data Availability

The original contributions presented in the study are included in the article, further inquiries can be directed to the corresponding authors.
